# Water, Sanitation, and Hygiene in Schools in Low Socio-Economic Regions in Nicaragua: A Cross-Sectional Survey

**DOI:** 10.3390/ijerph120606197

**Published:** 2015-05-29

**Authors:** Tania Jordanova, Ryan Cronk, Wanda Obando, Octavio Zeledon Medina, Rinko Kinoshita, Jamie Bartram

**Affiliations:** 1 Department of Environmental Sciences and Engineering, The Water Institute, University of North Carolina, Chapel Hill, NC 27599, USA; E-Mail: rcronk@live.unc.edu; 2 The United Nations Children’s Fund (UNICEF), Managua 10000, Nicaragua; E-Mails: wobando@unicef.org (W.O.); rkinoshita@unicef.org (R.K.); 3 Independent Consultant, Managua 10000, Nicaragua; E-Mail: octaviozmedina@yahoo.es

**Keywords:** equity, monitoring and evaluation, Sustainable Development Goals, WaSH

## Abstract

Water, sanitation, and hygiene (WaSH) in schools contributes to better health and educational outcomes among school-aged children. In 2012, UNICEF Nicaragua and partners conducted a cross-sectional survey of WaSH in 526 schools in 12 low socio-economic status municipalities in Nicaragua. The survey gathered information on: school characteristics; teacher and community participation; water and sanitation infrastructure; and hygiene education and habits. Survey results were analyzed for associations between variables. WaSH coverage was significantly higher in urban than rural areas. Presence of drinking water infrastructure (43%) was lower than sanitation infrastructure (64%). Eighty-one percent of schools had no hand washing stations and 74% of schools lacked soap. Sanitation facilities were not in use at 28% of schools with sanitation infrastructure and 26% of schools with water infrastructure had non-functional systems. Only 8% of schools had budgets to purchase toilet-cleaning supplies and 75% obtained supplies from students’ families. This study generates transferable WaSH sector learnings and new insights from monitoring data. Results can be used by donors, service providers, and policy makers to better target resources in Nicaraguan schools.

## 1. Introduction

Access to water, sanitation, and hygiene (WaSH) in schools is important for human health and well-being. Some of these benefits have yet to be fully achieved in low- and middle-income countries, due to low WaSH coverage. Proposed targets and indicators for the Sustainable Development Goals (SDGs) seek universal access to WaSH in non-household settings, such as schools and health care facilities [1,2,3,4]. Inadequate WaSH in schools results in adverse health outcomes among children including infectious, gastrointestinal, neuro-cognitive and psychological illnesses [5]. Inadequate WaSH conditions have been reported to reduce educational outcomes in children by contributing to absenteeism [6,7,8,9] and impaired cognitive abilities [10,11,12]. Bartlett summarized the impact of unsanitary conditions and diarrheal disease on child malnutrition and mental and social development, including IQ, school achievement levels, working memory, and behavioral problems [11]. The quality of sanitation facilities in schools can impact attendance rates of girls, especially once they have started to menstruate [13], while a more recent literature review found inconclusive evidence on the subject [14].

Improving WaSH in schools is linked to multiple benefits. A national study in India using longitudinal data demonstrated that latrine provision resulted in increased enrollment and a higher number of students passing exams [15]. Broader impacts on communities are reflected in studies such as that of O’Reilly *et* *al.* who identified that 14% of parents reported treating their water after a school-based WaSH intervention, compared with 6% at baseline, suggesting that children can transfer hygiene behaviors learned at schools to their households and to other members of their family [7,9,16]. 

Because of these health and educational benefits, national governments and multilateral agencies such as the United Nations Children’s Fund (UNICEF) have emphasized frequent monitoring of WaSH key indicators in schools to target resources and programmatic interventions to improve coverage [2]. In Nicaragua, a low-income country in Latin America, national actors including the Ministries of Education and Health and international donors have partnered to improve WaSH in schools by incorporating the Child-Friendly and Healthy Schools Initiative (CFHS), an initiative that provides a comprehensive protective environment to support quality education, into national policy [17]. While Nicaragua has shown a commitment to improving WaSH in schools by adopting these policies, a basic monitoring system on WaSH in schools in Nicaragua still needs improvement, especially in collecting disaggregated data at the local level. 

In 2012, UNICEF Nicaragua collaborated with the Ministry of Education (MINED), regional and municipal Secretariats of Education, and the Regional Government’s Directorate for WaSH to conduct a survey of WaSH in schools in 12 municipalities. The objectives were to: assess WaSH conditions in schools (to identify priority needs and to provide a baseline from which to measure the effectiveness of interventions); and analyze associations between variables (to determine possible solutions for improving conditions and informing future planning, programs, and research).

## 2. Methods

### 2.1. Study Area

This study took place in 12 municipalities of the North and South Atlantic Caribbean Coastal Regions (RACCN and RACCS respectively) of Nicaragua. The RACCN and RACCS are home to the largest proportion of indigenous and afro-descendent populations in the country. They also have some of the worst social and economic conditions in the country, especially indicators for multi-dimensional child poverty [17].

### 2.2. Study Design, School Sampling, and Data Collection 

Data were collected through a 102-question survey distributed to school directors. The survey was based on questions from the UNICEF *WaSH in schools: package of tools for monitoring* [18] and also incorporated lessons learned from other Latin American countries that conducted similar surveys [19]. Questions were further refined to account for subnational (regional) context based on input from MINED and the Regional Secretariats of Education. The survey was pre-tested in education evaluation meetings with the delegates and further pilot tested at a school in Bluefields, RACCS. The survey was intended to be a census of school-based data targeting all 1229 schools identified by MINED. These 1229 schools included 631 schools registered with MINED, along with informal schools taught by volunteer teachers organized through churches or community collectives.

Data were collected between November and December 2012. MINED municipal delegates delivered questionnaires to school directors in each municipality, including to non-MINED schools. Delegates from two out of five municipalities surveyed in RACCN did not attend training meetings. In these cases, UNICEF consultants visited municipalities to ensure that surveys were distributed and collected based on study guidelines. Delegates were given 30 days to collect and return the completed questionnaires at the next monthly education meetings. Completed surveys were reviewed by surveyors, entered into a database for analysis and inconsistent responses were confirmed via telephone.

### 2.3. Variables and Data Analysis

The continuous variables in the survey were the numbers of students, teachers, classrooms, toilets, and sinks at each school, and number of years since the most recent renovations at each school. All other variables were categorical. Continuous variables were normalized using ratio calculations such as a student-to-toilet ratio. This ratio was based on the total number of toilet seats at the school for male and female students and teachers. Categorical variables were primarily binary. Variables with more than three categories were aggregated into two or three bins to increase bin size and assist in data analysis.

Schools were categorized into three groups based on residential density of their location: (1) urban, (2) rural village, and (3) low density rural. School grade levels were preschool, primary, secondary, and post-secondary; with some schools having more than one grade level category. There were very few post-secondary schools in the study sample and these were excluded from the analysis. Over 94% of schools with preschool grade levels also had primary grade levels. Thus schools with preschool and primary and those with only primary grade levels were aggregated into one category, “primary”. Schools with both primary and secondary levels were coded under the category of “all levels”. The survey asked questions about the condition, operation, and maintenance of water and sanitation infrastructure. The grouping and coding of select questions is summarized in Table 1. 

**Table 1:** Coding of selected questions.

Questions	Responses	Coded Binary Variables
**“Does your school have a water system?”**	(a) Yes, the school has a functioning water system. (b) No, the school doesn’t have a water system, and no one carries water to school. (c) No, the school doesn’t have a water system, but water is carried to school. (d) Yes, the school has a water system, but it is broken and no one carries water to school. (e) Yes, the school has a water system, but it is broken and water is carried to school.	(1) schools with water infrastructure (a, d & e) (0) schools without (b & c)
		(1) schools with access to water (a, c & e) (0) schools without access (b & d)
		For schools without functioning water system: (1) water carried to school (c & e) (0) water not carried (b & d)
**“Does your school have sanitation infrastructure?”**	(a) Yes, there is sanitation infrastructure and it is being used. (b) Yes, there is sanitation infrastructure, but it is not in use. (c) No, there is no sanitation infrastructure at the school.	(1) schools with sanitation infrastructure (a & b) (0) schools without (c)
		For schools with san. infrastructure: (1) infrastructure in use (a) (0) not in use (b)
**“How often are sanitation facilities cleaned?”**	(a) two times per day or more (b) one time per day (c) every other day (d) one time per week (e) less than once per week	(1) cleaned several times per week (a, b & c) (0) once per week or less (d & e)
**“Is there a dedicated budget at the school for the purchase of toilet cleaning supplies?”**	(a) Yes, there is a budget dedicated for cleaning supplies. (b) There is no dedicated budget, but student families donate money/supplies. (c) No, cleaning is only done sporadically, through the aid of environmental brigades, community organizations, nonprofits, *etc.*	(1) school budget for toilet cleaning supplies (a) (0) no budget for toilet cleaning supplies (b & c)
**“Is there a dedicated budget at the school for the purchase of soap, paper towels, *etc.*?”**	(a) Yes, there is a budget dedicated for hand washing materials. (b) There is no dedicated budget, but student families donate money/materials. (c) No, hands are only washed with water. (d) No, hands are not washed at the school. (e) Other.	(1) school budget for hand washing materials (a) (0) no budget for hand washing materials (b, c & d)
		Of schools where hands are washed: (1) hands washed with soap (a & b) (0) washed with water only (c)
**“Are hygiene training requirements included in the school action plan?”**	(a) Yes. (b) No, they are not included. (c) No, there is no School Action Plan.	(1) hygiene component in School Action Plan (a) (0) no component in School Action Plan (b & c)
		(1) School Action Plan (a & b) (0) no School Action Plan (c)
**“Does your school have a general hygiene awareness program?”**	(1a) Yes. (1b) No.	(1) at least one type of hygiene training program in the past three years (1a, 2a, 2b, 2c, 3a,3b, or 3c) (0) no hygiene training programs in past three years (1b, 2d, & 3d)
**“Has your school implemented an environmental health training in the past three years?”**	(2a) Yes, there have been separate workshops for teachers and students. (2b) Yes, workshops for teachers, which have been replicated to students. (2c) Yes, workshops for students only. (2d) No, there has been no training on this topic.	
**“Has your school implemented a personal hygiene training in the past three years?”**	(3a) Yes, there have been separate workshops for teachers and students. (3b) Yes, workshops for teachers, which have been replicated to students. (3c) Yes, workshops for students only. (3d) No, there has been no training on this topic.	

As shown in Table 1, the survey investigated three hygiene education programs at the school: (1) a general hygiene awareness program; (2) environmental health training activities for teachers or students in the past three years, and (3) personal hygiene training activities for teachers or students in the past three years. In addition to analyzing these three variables separately, they were also combined into one binary variable of (a) schools which had at least one type of hygiene training program in the past three years and (b) schools which had no such programs. Additionally, the survey asked whether hygiene training was being implemented using the Healthy Families, Schools, and Communities (FECSA) method. This method was developed by the Nicaragua Ministry of Health to be implemented in all formal and informal schools in order to improve public health in communities.

Survey data were analyzed using R version 2.15.2 [20]. Chi-Square tests were used to identify relationships between two categorical variables. Chi-Square tests for equality of proportions were used when at least one of the categorical variables had two levels, while tests for independence of all factors were used when both categorical variables had more than two levels. Logistic regression was used to examine relationships between variables when the outcome variable was binary, but the explanatory variable was continuous or when more than one explanatory categorical variable was included in the model. Results of the significant logistic regression models are summarized with odds ratios and 95% confidence intervals. All logistic regressions reported in this study controlled for urban-rural location and school size (*i.e.*, number of students), unless otherwise stated.

While there are some socio-economic and health data available for Nicaragua at the municipal level, no secondary data source at the school-level existed at the time of analysis. Thus, no additional school- and community-level correlates or control variables, beyond the questions asked in the survey, could be analyzed. Urban-rural setting of the school, as determined by one survey question, is often correlated with community-level socio-economic variables and WaSH conditions, and thus was used as a control during statistical analyses.

Additionally, due to the lack of information about individual schools, the characteristics of the schools which did not respond to the survey were unknown. Thus no additional analyses could be conducted to test whether survey respondents were systematically different from non-respondents.

### 2.4. Ethics Statement 

Data collection methods were submitted to the University of North Carolina at Chapel Hill Institutional Review Board (IRB) for approval. This study was determined to not constitute human subjects research and received an exemption from the IRB under study number 14-1408. 

## 3. Results

### 3.1. General Characteristics 

**Figure 1:**
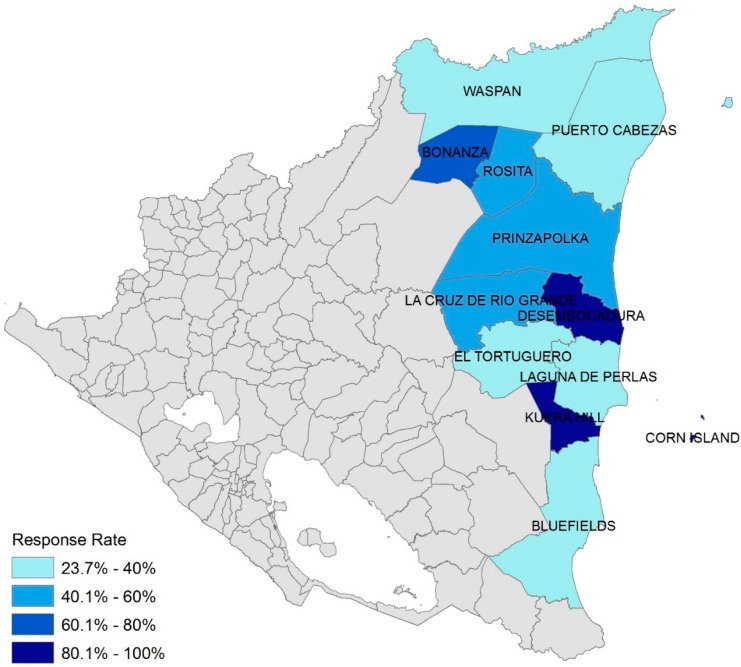
Survey response rate by municipality.

Surveys were completed and returned by 526 schools (43% response rate). These included: 10 preschool, 162 preschool and primary, 255 primary only, 36 secondary only, 42 with all levels, and 31 unspecified schools. The returned surveys had varying levels of completeness, with lower response rates for certain municipalities and questions. Whenever a percentage is reported, the number of respondents is provided for reference either in the text or in a table. Figure 1 shows the geographic location of the survey municipalities and indicates the response rate by municipality, with darker colors representing higher response rates. The survey responses from areas with low response rates may not be representative of the entire school population.

Primary schools accounted for 83.7% of survey responses, while secondary schools accounted for 6.9%, and schools including both primary and secondary levels made up 9.4% of responses. Based on survey results, schools in low density rural areas were predominantly primary schools (95%), with rural village areas having 73% and urban having 65% primary schools (n = 491, *p* < 0.001). 

Table 2 and Table 3 indicate that there are substantial differences between schools in urban, rural village, and low density rural areas. Schools in rural areas tended to be newer, have fewer students and classrooms, and were mostly primary schools. For example, urban areas had larger schools with a median of 282.5 students and 6 classrooms, while rural village (median = 70 students, 2 classrooms) and low-density rural areas (median = 32 students, 1 classroom) had much smaller schools with fewer students.

Rural village and low density rural areas had a higher proportion of schools aged between 0–10 years as compared to urban areas. In these rural areas, the older the school building, the more likely it was to have water infrastructure: 64% of schools more than 20 years old had water infrastructure, while 53% of schools 11–20 years and 27% of schools 0–10 years old had water infrastructure (n = 345, *p* < 0.001). In urban areas, the school buildings in the middle age range (11–20 years old) had the lowest percentage of water infrastructure. In low density rural areas, the school buildings aged 0–10 years had significantly fewer sanitation facilities (49%) than schools more than 10 years old (69%), (n = 220, *p* < 0.01).

**Table 2:** School characteristics based on survey responses.

Factor		Low Density Rural		Rural Village		Urban		Total
		%	n *	%	n *	%	n *	
*Total Schools by Urban-Rural Setting **^†^***		53%	267	29%	148	17%	87	502
*School Type*	Primary	95%	250	73%	108	65%	53	411
	Secondary	2%	6	10%	15	16%	13	34
	Multiple/All Grade Levels	2%	6	16%	24	20%	16	46
*School Building Age*	0–10 Years	67%	172	46%	60	40%	32	264
	11–20 Years	25%	65	37%	48	22%	18	131
	>20 Years	8%	21	18%	23	38%	31	75
*School Size*	Median (Mean) # of Students (n = 239)	23.8 (27.3)		30.0 (46.3)		55.2 (67.6)		27.4 (40.0)
	Median (Mean) # of Classrooms (n = 392)	1 (1.55)		2 (3.13)		6 (6.58)		2 (2.86)

**^†^** Note: 24 schools did not specify urban-rural setting. * The n columns represent the number of samples from which the percentage to the left is derived.

**Table 3:** Urban-rural patterns in select variables of wash in schools.

Factor		Low Density Rural		Rural Village		Urban		Total	*p*-Value ^†^
		% Yes	n *	% Yes	n *	% Yes	n *		
Water in Schools									
	Water Infrastructure at School (working and damaged)	28%	230	58%	137	68%	75	442	**<0.001**
	Of schools w/water infrastructure, % functioning	77%	64	65%	79	82%	51	194	**0.062**
	Of schools w/damaged water infrastructure, % carry	40%	15	46%	28	44%	9	52	0.921
	Of schools w/o water infrastructure, % carry	47%	166	34%	58	38%	24	248	0.213
	Functioning Water Infrastructure	21%	230	37%	137	56%	75	442	**<0.001**
	Water Carried to Schools (in case no water infrastructure or damaged)	37%	230	24%	137	17%	75	442	**0.002**
	No Access to Water (in case no water infrastructure or damaged)	42%	230	39%	137	27%	75	442	0.056
	Water Treatment	50%	101	71%	58	83%	36	195	**<0.001**
	Water Filter for Rain Water	28%	53	21%	43	15%	26	122	0.405
	Water Inspections by Government Officials	9%	128	21%	82	27%	52	262	**0.004**
	Students Drink from a Shared Cup	48%	129	37%	79	34%	38	246	0.151
Sanitation in Schools									
	Sanitation Facilities	56%	226	73%	113	79%	62	401	**<0.001**
	Sanitation Facilities Used	70%	126	67%	82	82%	49	257	0.183
	Disuse due to: Poor Conditions	44%	55	52%	46	57%	14	115	0.446
	Disuse due to: Latrines Full	36%	55	24%	46	21%	14	115	0.446
	Disuse due to: Not accustomed	15%	55	9%	46	7%	14	115	0.446
	Toilets with Lid	19%	154	23%	113	35%	68	335	**0.038**
	Gender Separated Toilets	24%	165	44%	119	49%	72	356	**<0.001**
	Latrines Cleaned Several Times/Week	35%	144	32%	95	55%	58	268	**<0.01**
*Hygiene in Schools*									
	Handwashing Stations	11%	239	25%	139	32%	74	452	**<0.001**
	Students Wash Hands w/ Soap	22%	183	25%	111	42%	57	351	**0.01**
	General Hygiene Awareness Program	35%	209	52%	133	65%	69	411	**<0.001**
	Environ. Health Training (past 3 years)	19%	210	21%	128	64%	69	407	**<0.001**
	Personal Hygiene Training (past 3 years)	22%	215	29%	126	54%	69	410	**<0.001**
	Training follows FECSA Methodology	10%	194	11%	115	13%	69	378	0.822
	No Training Program in Past 3 Years	54%	203	33%	126	18%	71	400	**<0.001**
	General School Action Plan	60%	206	68%	101	91%	67	374	**<0.001**
	Hygiene in School Action Plan	56%	124	78%	69	77%	61	254	**0.001**
*Community Involvement*									
	Parent Association	93%	258	87%	141	94%	80	479	0.080
	Teacher Association	23%	222	30%	123	51%	70	415	**<0.001**
	Student Association	59%	248	56%	130	76%	75	453	**0.012**
	Parents Participate in WaSH in School	30%	142	47%	86	31%	52	280	**0.034**
	Teachers Participate in WaSH in School	31%	131	60%	75	50%	46	252	**<0.001**

* The n columns represent the number of samples from which the percentage to the left is derived. **^†^** *p*-values are derived from chi-sq. tests between each variable and the urban-rural location variable. Thus, the table includes as many tests as there are rows.

### 3.2. Water in Schools 

Less than half of schools had access to water infrastructure (piped connections, boreholes, and wells with hand pumps or electric pumps) (43%, n = 456). As shown in Table 3, the percentage of schools with water infrastructure varied significantly between urban and rural locations, with 28% in low density rural areas, 58% in rural villages and 68% in urban areas (*p* < 0.001). Carrying water to the school was the most common type of water access in low density rural areas. 

Of the 197 schools with water infrastructure, 52 (26%) reported that the water system was non-functional (*i.e.*, damaged or not functioning properly). The highest percentage of non-functional water systems was in rural villages (35%), while low density rural areas had 23% non-functional water systems, and urban areas had 18% non-functionality. Thus, when accounting for water system functionality, 32% of schools had functional water systems, 30% had water carried to school from an outside source, and 38% had no access to water (n = 456).

We conducted a logistic regression to test whether infrastructure age was associated with functionality. Not surprisingly, the results showed that of schools with water infrastructure, newer water infrastructure (0–5 years in age) was more likely to be functioning than older infrastructure (over 5 years in age), (OR 3.4; 95% CI: 1.2–10.0; *p* = 0.02). A separate logistic regression showed that the odds that water infrastructure was functioning at a school with teacher involvement in water infrastructure planning and maintenance were 2.3 (95% CI: 0.97–5.4; *p* = 0.0599) times the odds at a school without teacher involvement, when urban-rural location was controlled for, though the result was narrowly not significant. When age of infrastructure is added to the logistic regression model, teacher involvement is even less statistically significant. 

School directors were asked whether water at the school was treated prior to consumption by students, by a clay filter, chlorination, the SODIS method, any other method, or no treatment. Of the 77 “other” responses, 68 were “I don’t know”. This means that 24% of respondents were uncertain or did not know if the water at their school was treated. For this reason, the “other method” category was excluded from analysis. Water treatment followed a similar urban-rural pattern as water infrastructure, ranging from 50% of schools in low density rural areas to 71% of schools in rural villages and 83% of schools in urban areas that had water treatment (n = 195, *p* < 0.001). A separate question asked directors at schools where rain water was collected, whether that water was filtered or purified. Of the 134 respondents, 29 (22%) responded that a filter or purification was used. 

School directors provided information on whether health authorities had tested the school water. Government health inspections of school water quality also follow an urban-rural trend, with urban schools and schools with water infrastructure being inspected more than schools in low-density rural areas. Based on school director responses, water quality had been tested by government health officials at 27% of urban schools, 21% of rural village schools, and 9% of low density rural schools (n = 262, *p* = 0.004). These water quality data were not available for analysis in this article.

Children may drink water from a shared cup while at school. Though not statistically significant, the percentage of schools where survey respondents reported that students drink from a shared cup varied between 48% in low density rural areas and 34% in urban areas (*p* = 0.151).

### 3.3. Sanitation in Schools 

In the study area, 64% of schools had improved sanitation facilities on premises (n = 412), but 28% of schools with sanitation facilities reported that they were not used. School directors reported that facilities were not in use due to poor conditions (47%), full latrines (29%), people not accustomed to using them (13%) and other reasons (11%) (n = 118). Based on a logistic regression, schools with sanitation infrastructure 0–5 years in age were more likely to have sanitation infrastructure that was in use than schools aged 6–10 years (OR 2.4; 95% CI: 1.2–5.1; *p* < 0.05) and even more so than schools over 10 years in age (OR 3.7; 95 CI: 1.6–8.9; *p* < 0.01).

Of schools with toilets, 19%, 23% and 35% had lids (n = 335) in low density rural, rural village, and urban areas respectively and 5% of all schools had toilet paper (n = 327). Of schools with sanitation, the majority, 70%, had water-less latrines, 17% of schools had a latrine system using water, 8% used a septic-system, 2% were connected to a community sewer system, and 3% reported “other” (n = 307). 

Following patterns in water infrastructure, the percentage of schools with sanitation infrastructure exhibited rural-urban differences with coverage of 56% in low density rural, 73% in rural village, and 79% in urban schools (n = 401, *p* < 0.001). The percentage of schools with gender-separated toilets was significantly lower in low density rural schools (24%) than in rural village schools (44%) and urban schools (49%) (n = 356, *p* < 0.001). Of the schools with sanitation facilities, 52% of low density rural schools (n = 126) had only one toilet, as compared to 22% in rural village schools (n = 76) and 15% in urban schools (n = 47).

**Figure 2:**
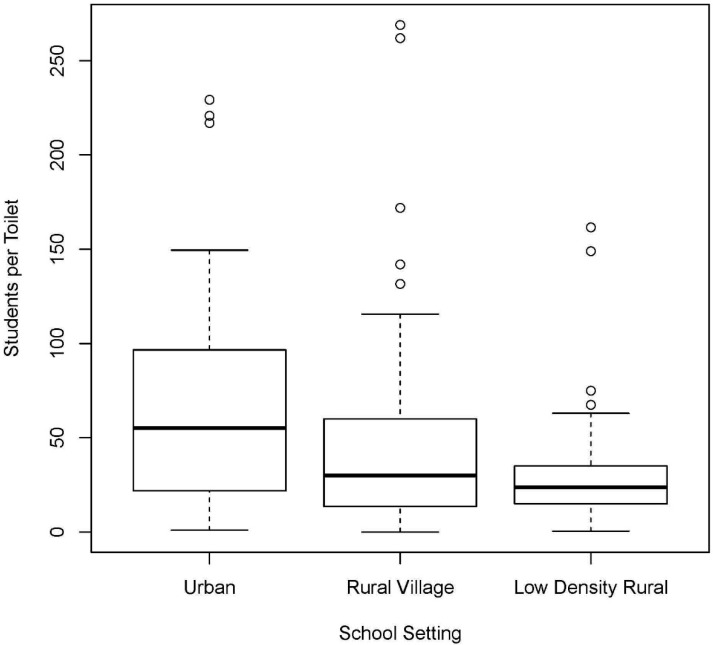
Student to toilet ratio for schools with at least one toilet, by school setting.

Urban areas had significantly higher student-to-toilet ratios than rural areas, as shown in Figure 2. These ratios are based on the number of toilet seats at each school. In schools with at least one toilet, the median student to toilet ratio was 55 for urban, 30 for rural village, and 24 for low density rural areas. Based on MINED policy, Nicaragua’s standard is a maximum of 30 students per toilet [21]. Thus, based on the median, schools in urban areas did not meet this standard. However, this ratio does not include schools without sanitation infrastructure which also do not meet Nicaragua’s school sanitation goals. If schools without any toilets are included in the calculation, the median student to toilet ratio was 73 for urban, 66 for rural village, and 63 for low density rural areas. The student-to-toilet ratio was lowest in primary level only schools. There was no significant difference in the percentage of schools with toilets separated by gender between secondary and primary schools, after controlling for rural-urban location.

A higher proportion of urban schools had cleaning of sanitation facilities several times per week, while rural areas had a higher proportion of schools with once per week or less frequent cleaning (*p* < 0.01). Toilet facilities were cleaned several times per week in 55% of urban schools and 32% and 35% of rural village and low density rural schools, respectively (n = 297). Irrespective of urban-rural location, more frequent sanitation facility cleaning was associated with personal hygiene education within the past three years at the school, presence of a teachers association, and school cleaning supply budget. Though narrowly not significant, the odds of a school with personal hygiene training having sanitation facility cleaning several times per week were 1.8 (95% CI: 0.99–3.3; *p* = 0.053) times the odds of a school without such training. Additionally, schools with teachers associations were more likely to have sanitation facility cleaning several times per week than schools without a teachers association (OR 1.8; 95% CI: 1.0–3.2; *p* < 0.05). Finally, schools with a dedicated cleaning supply budget were more likely to have sanitation facility cleaning several times per week than schools without such a budget (OR 1.2; 95% CI: 1.03–11.1; *p* < 0.05). Of the 224 schools with cleaning supplies for sanitation facilities, only 10% had a dedicated budget, while 90% of schools had supplies donated by the families of students.

### 3.4. Hygiene in Schools 

The survey investigated the implementation of three separate hygiene training programs at schools. The proportion of schools implementing each of the three programs differed significantly between urban and rural locations. The most significant disparities were noted on whether a school had environmental health training in the past three years. Only 19% of low density rural and 21% of rural village schools had such a program, as compared to 64% of urban schools (n = 407, *p* < 0.001). Personal hygiene training at the school within the past three years followed a similar pattern, with such a program being present in 22% of low density rural, 29% of rural village and 54% of urban schools, respectively (n = 410, *p* < 0.001). During the 2012 school year, 35% of low density rural, 52% of rural village and 65% of urban schools implemented a general hygiene awareness program for students and teachers at the school (n = 411, *p* < 0.001). However, 288 out of 389 responding schools (74%) had never heard of the FECSA methodology, and of the 196 schools with hygiene programs, only 22% were using FECSA. The proportion of schools using the FECSA methodology did not differ between rural and urban areas. Overall, 41% of schools did not implement any of the hygiene programs discussed in the survey (n = 414). This does reflect urban-rural discrepancies (Table 3). Fifty-four percent of low density rural schools lacked a hygiene program compared to 33% of rural village and 18% of urban schools without a program. (n = 400, *p* < 0.001).

Logistic regression indicated that schools with a personal hygiene training in the past three years were more likely to have teacher participation in water infrastructure planning and maintenance as compared to schools without a personal hygiene training (OR 2.3; 95% CI: 1.3–4.3; *p* < 0.01). However, environmental health training and a general hygiene awareness program were not significantly associated with teacher participation.

While 91% of urban schools had a general school action plan (not hygiene-specific), this percentage was lower in rural villages (68%) and only 60% in low density rural areas (n = 374, *p* < 0.001). Of the 254 schools with action plans, 77% of urban and 78% of rural village schools included a component on hygiene training in the action plan. In contrast, 56% of low density rural schools included this component in their action plan (*p* < 0.01). Schools with a hygiene component in the school action plan were more likely to implement at least one type of hygiene training program in the past three years than schools without a hygiene component in the action plan (OR 3.8; 95% CI: 2.0–7.6; *p* < 0.0001).

Hand washing practices were limited at the surveyed schools: 81% of schools did not have hand washing stations (n = 464) and of schools where hands were washed, 74% washed hands without soap (n = 362). Soap availability differed between rural and urban schools. Forty-two percent of urban schools had a budget or community support to provide soap, as compared to 25% of rural village and 22% of low density rural schools (n = 350, *p* < 0.01). Of the 95 schools with soap, 95% acquired the soap from parents of students, rather than through a dedicated budget.

### 3.5. Community Involvement in Schools 

This survey examined the existence of parent, teacher and student associations at the schools and whether they were involved with WaSH operations and maintenance, since community involvement may play a role in providing WaSH in schools. Overall, 91% of schools had a parent association, 61% had a student association, and 29% had a teacher association. Urban-rural variations were significant for student associations, with 56% of rural village areas, 59% of low density rural areas and 76% in urban areas having student associations (n = 453, *p* = 0.012). Differences were also significant for teacher associations, with 23%, 30% and 51% of schools having such associations in low density rural, rural village and urban locations, respectively (n = 415, *p* < 0.001). 

Based on logistic regressions, the odds that a school with a teachers association had water infrastructure were 2.9 (95% CI: 1.7–5.0; *p* < 0.0001) times the odds for a school without a teachers association. Similarly, the odds that a school with a teachers association had sanitation infrastructure were 1.8 (95% CI: 1.0–3.3; *p* < 0.05) times the odds for a school without a teachers association.

There was no difference in the level of participation of parents in the planning and/or maintenance of water systems between urban (31%) and low density rural schools (30%), while rural village areas had a significantly higher percentage (47%, n = 278, *p* = 0.037). Teachers had the highest involvement in planning, implementation or monitoring of water system maintenance in rural village areas, which also had the highest parent participation. The percentage of schools with teacher involvement ranged: 31%, 60%, and 50% in low density rural, rural village, and urban locations, respectively and this was statistically significant (n = 252, *p* < 0.001).

There were two variables associated with teacher involvement in water system planning or maintenance. Logistic regression indicated that the odds of a school with a teachers association having teacher involvement in water infrastructure planning and maintenance were 3.24 (95% CI: 1.7–6.1; *p* < 0.0001) times the odds of a school without a teachers association. Additionally, the odds of a school with at least one type of hygiene awareness program having teacher involvement in water infrastructure planning and maintenance were 2.1 (95% CI: 1.1–3.9; *p* < 0.05) times the odds of a school without any hygiene awareness programs.

## 4. Discussion

The findings of this study are consistent with evidence on WaSH in schools in other countries and highlight several common WaSH in schools challenges. This study provides evidence of the low WaSH in schools coverage in Nicaragua’s RACCN and RACCS regions. The analysis of WaSH in schools data from disadvantaged regions of Nicaragua indicates that water coverage at schools was significantly lower than sanitation coverage, across both urban and rural areas. Conditions are likely worse than specified in the survey. For example, not all improved water sources are necessarily free of fecal contamination, so access to “safe” water coverage may in fact be lower than reported in this survey since water quality testing was not conducted [22,23]. Access to water services and the quality of water varies by season and water quality is typically worse in the wet season [24]. Stored water quality is generally much worse than water from the source leading to further contamination that was not considered in this analysis [25].

In low density rural areas with no direct access to water at school, many parents and students assist with bringing water to the school from other locations. Additionally, survey results showed that over 90% of sanitation cleaning supplies and soap for hand washing, were contributed by student families. Parents, and possibly the broader community, may be able to play an important role in addressing WaSH needs in schools. When school budgets do not allow for improvement in WaSH in schools, parents might be able to provide resources to ensure a healthy and safe school environment. However, such a strategy also puts an undue burden on poor communities to pay extra, and can thus act to further increase inequality and undermine sustainability. 

In terms of sanitation, most urban schools and half of rural village schools did not meet the national student-to-toilet standard. To improve this ratio, implementers might focus their efforts on building more urinals for boys which are cheaper than toilet blocks, are longer lasting, and allow more resources to be used for building latrines for girls [26]. Toilet blocks separated by gender are low (ranging from 24% in low density rural to 49% in urban areas). This may result in girls not attending school due to lack of privacy when using the toilet, lack of cleaning materials, lack of soap for hand washing and when menstruating [27]. Other studies have also found that pubescent girls are going to school in environments that are not gender sensitive and lack adequate facilities and supplies [28]. An investment in separate latrines for girls may boost girls’ attendance [8,15].

A lack of hand washing infrastructure and resources in schools also has adverse effects on school-aged children. Lack of water and soap for hand washing can increase the spread of diseases among students. We found that 81% of schools did not have hand washing stations and students at 71% of schools washed their hands solely with water. It should be noted that the presence of hand washing facilities does not necessarily indicate a higher prevalence of hand washing [29]. Nonetheless, studies indicate that the potential for long-term behavior change in students to form a sustained habit of properly washing hands is undermined by unreliable availability of soap and water [30,31].

Another challenge identified by survey respondents was maintenance. Survey data showed that older water and sanitation systems were less likely to be functional than newer infrastructure. Of schools that reported that toilets were not in use, the most common reason given was poor conditions, while the second most common reason was full latrines. These two reasons are related to the maintenance of sanitary facilities, which has been shown to have an impact on school absence. For example, in a cross-sectional study in Kenya, children who attended primary schools with better maintained latrines were less likely to have had a recent absence [32]. We found three factors that were associated with sanitation facility cleaning several times per week: personal hygiene training at the school, teachers association at the school, and a school cleaning supply budget. Similarly, Chatterley *et* *al.* (2014) found that conditions needed for the sustainable management of sanitation facilities in Bangladeshi schools included not only financial support and quality construction, but also “incentivizing conditions,” such as a sanitation champion, a teacher with roles and responsibilities for sanitation maintenance, and/or school management committee involvement in sanitation [33]. Recommended actions from a study in Ethiopia on the impact of WaSH in schools on learning included: “establishing effective school sanitation clubs, raising parent awareness through education, (and) strengthening sector collaboration and advocacy on relationships between education and WaSH” [34].

This survey included questions to assess some of the six enabling environment domains for sustainability of WaSH in schools: financial capacity; accountability; technical feasibility and availability; community support; school leadership and management; and student engagement [35]. In terms of school leadership and management, results indicated that a hygiene component in the school action plan was associated with at least on hygiene training program at the school. Additionally, a teachers association at the school was significantly related to presence of water and sanitation infrastructure, sanitation facility cleaning several times per week, and at least one hygiene training program at the school. 

Results also indicated that teacher involvement in school water planning and maintenance was associated with the presence of a teacher association or a hygiene program at the school. While more evidence is necessary to identify the causal pathway of this relationship, the results could provide insight into how teacher involvement in WaSH in schools can be increased. For example, the presence of a teachers association can provide the organizational structure and peer support to establish a school water and sanitation management committee. Alternatively, hygiene training at the school can increase teacher awareness of the importance of water and sanitation maintenance and spur participation in such activities, and vice versa. The potential benefits that teachers, parents and community members can contribute to WaSH in schools, as well as the mechanisms of such contribution, should be studied further, both in Nicaragua and within other cultural contexts in other countries. 

Inequalities in WaSH access persist between urban and rural regions and at sub-national levels which is consistent with studies in other countries on inequalities in access to WaSH, including those on water access in schools [36,37]. Water and sanitation systems in rural areas tended to be less functional than those in urban areas, also urban areas had better access to WaSH operations and maintenance [38,39]. There are several possible reasons for low coverage and inequalities in coverage. These include insufficient funds and/or inadequate allocation of school budget to provide WaSH in schools, insufficient funds for operations and maintenance and the difficulty of maintenance technicians to reach low density rural schools. Future studies and monitoring efforts might explore the relationship between sustainability factors and the role of the enabling environment in maintaining sustainability [40].

### 4.1. Priorities for Monitoring 

This survey was intended to gather baseline information on WaSH at the school level, as there was a lack of systematic and standardized data reporting from schools to regional and national governments. Because of the lack of a system of monitoring and surveillance, the government and donors may not have been aware of the low WaSH coverage and urban-rural disparity in the access and quality of WaSH in schools in these regions that the survey revealed.

One example of a lack of data was that schools did not regularly (*i.e.*, at fixed time intervals) nor frequently report student attendance to municipal MINED offices. Thus, the survey requested that school principals estimate monthly student absences due to health. As most schools did not keep records on absence rates, the estimates might have been inaccurate. While we found a significant statistical relationship between prolonged student absences from school that lacked water infrastructure, we did not report these findings due to the unreliability of the variable. A future study with more accurate tracking of reasons for school absences may assist in determining the validity and strength of a relationship between WaSH in schools and student absences.

Two key issues of survey design emerged from our analysis: question validity and questions with the potential for response bias. Validity is the degree to which the question measures what it claims to measure, while response bias can occur in self-reported surveys due to participants’ beliefs and motivations which may produce inaccurate or untruthful responses. This survey included questions that were important to assess WaSH in schools, but were not summarized in this article due to these issues of reliability. In addition to student absences, excluded variables included one on water quality, one on community involvement, and two on student and teacher hand washing habits.

For example, survey results showed that 82% of school directors reported that students had a habit of washing hands while at school at least once per day. However, this is not likely indicative of actual hand washing rates, since self-report of hand washing behavior has been shown to be biased and consistently produce much higher rates than those reported from observed behavior [29]. Alternatively, directly observing hand washing behavior can be intrusive, expensive, and has potential for observation bias [29]. In response to these issues, studies have pointed to the need for the use of “leading indicators”, that are less susceptible to response bias, when monitoring WaSH in schools [29,34]. 

Additionally, this survey asked school directors to take a water sample and describe its physical characteristics (e.g., smell, color, and particulates). However, these are considered invalidated measures of microbial water quality. Instead, microbial water tests by trained individuals at random samples of schools could provide more accurate information on water quality. This survey indicated that very few schools had water quality tested by government officials. Increased resources or alternative actors for water quality monitoring should be considered.

While conducting a census of schools was ambitious, areas with low response rates might not have been accurately represented by the survey data. Future studies might use a random sample of schools and send enumerators to the school sites to spot check and validate WaSH conditions reported by questionnaires. One option could be to deploy “rapid surveys”, with fewer questions and a smaller sample size, as an alternative to long-format surveys and can be used to study a specific research question in a smaller geographic area. Studies might also use mobile devices as monitoring instruments to collect geospatial data points. With geolocation, monitoring data can be linked to other data sets to provide additional covariates for analysis which will provide further value and more robust analyses.

Monitoring allows the national government and donors to conduct program evaluation, planning, policy development and assess regulatory compliance [2]. Results of analysis of monitoring data can be used for advocacy to appropriately allocate a country’s resources and leverage the resources of donors and partners. A possible solution would be the widespread use of an educational management information system [18]. This system should incorporate standardized, robust indicators [2]. 

This study demonstrates the value from evaluation of monitoring data. First, we were able to obtain more value from monitoring data by using statistical tools to evaluate the data and identify significant findings for use by policy makers and practitioners. Second, it allowed us to understand impact and identify specific ways in which to improve performance. Third, evaluation of the monitoring data enabled us to derive learnings by identifying problems to drive further improvement in data collection. Though this survey was carried out in Nicaragua, the lessons learned on monitoring methodology can apply to data collection efforts in different regions around the world.

### 4.2. Limitations 

The original survey had a number of limitations. Self-reported surveys may suffer from reporting bias. Respondents may have reported a better WaSH status to give a favorable impression or reported worse conditions to attract resources for their school. The survey would have benefited from random spot check visitations by survey personnel. The survey instrument was pre-tested, with municipal representatives from educational agencies, rather than with school principals. There were cases where answers to certain questions contradicted answers of other questions, indicating a lack of understanding of all questions or low quality of data entry. 

Not all the questions were answered by all respondents. Not all of the survey questions aligned identically with internationally used indicators such as the improved/unimproved water source classification. There is a potential for non-response bias, as the response rate for the survey was 43%. The absence of disaggregated socio-economic, demographic, and geographic data for all 1229 schools that received the survey, prevented analysis to indicate whether the responding schools were representative of the entire school population. WaSH coverage may in fact be lower as harder-to-reach schools may not have responded. The lack of reliability and validity of certain questions, such as student absence rates due to illness, provided unusable data, and thus conclusions and recommendations for those subjects couldn’t be made. 

Despite its limitations, this study is one of the first sources of data revealing the status of WaSH in schools in disadvantaged regions of Nicaragua and one of the first sub-national studies on the status of WaSH in schools in the published literature. The study fills a major information gap, providing a preliminary baseline for more frequent monitoring. 

## 5. Conclusions 

School children need adequate and safe WaSH in schools to maximize health and educational outcomes. However, conditions in schools in the RACCN and RACCS regions of Nicaragua are insufficient and require further attention and improvement. There is potential for schools to focus on “quick win” solutions by enhancing less expensive activities, such as training and education programs for teachers and students. However, the sustainable impact of these solutions should also be considered. The national government, donors, international and national NGOs and other actors should prioritize investment in WaSH resources for schools, particularly for the most marginalized populations. Survey results can inform priorities for monitoring, practice, and improvements in WaSH in schools. Similar studies could be replicated in other countries or sub-national regions where there is a lack of quality disaggregated data on WaSH in schools, so that problems can be identified and resources can be targeted to improve health and educational outcomes in school-aged children. In addition to one-time studies, efforts should be made by national governments, in Nicaragua and other countries, to establish continuous monitoring systems to regularly track needs and improvements of WaSH in schools. This study offers insight and recommendations for designing future surveys and monitoring systems.
